# Evaluation of Intravenous Immunoglobulin in Pediatric Acute-Onset Neuropsychiatric Syndrome

**DOI:** 10.1089/cap.2020.0100

**Published:** 2021-03-15

**Authors:** Isaac Melamed, Roger H. Kobayashi, Maeve O'Connor, Ai Lan Kobayashi, Andrew Schechterman, Melinda Heffron, Sharon Canterberry, Holly Miranda, Nazia Rashid

**Affiliations:** ^1^ IMMUNOe Research Center, Centennial, Colorado, USA.; ^2^ Pediatric Immunology & Allergy, University of California, Los Angeles School of Medicine, Los Angeles, California, USA.; ^3^ Allergy, Asthma & Immunology Relief, Charlotte, North Carolina, USA.; ^4^ Midlands Pediatrics, Papillion, Nebraska, USA.; ^5^ Colorado Neurocognitive Consulting, Centennial, Colorado, USA.; ^6^ Dunwoody Consulting, Ventura, California, USA.

**Keywords:** IVIG, PANS, PANDAS, Octagam

## Abstract

***Objectives:*** Pediatric acute-onset neuropsychiatric syndrome (PANS) is a clinical diagnosis in children who have an acute manifestation of varied neuropsychiatric symptoms, including obsessive compulsive disorder, eating disorders, tics, anxiety, irritability, and problems with attention/concentration. PANS may develop as a result of a postinfectious syndrome and may represent a new form of postinfectious autoimmunity. To test the hypothesis that multiple, consecutive infusions of intravenous immunoglobulin (IVIG) for PANS can be efficacious, a multisite, open-label study was designed.

***Methods:*** The primary endpoint was evaluation of the efficacy of IVIG [Octagam 5%] in PANS over a period of 6 months (six infusions) based on mean changes in psychological evaluation scores using 6 different assessments, including the Children's Yale-Brown Obsessive Compulsive Scale (CY-BOCS), Clinical Global Impression of Severity, and the Parent-Rated Pediatric Acute Neuropsychiatric Symptom Scale (PANS Scale).

***Results:*** The final cohort consisted of 21 subjects (7 per site) with moderate to severe PANS. The mean age was 10.86 years (range: 4–16 years). Results demonstrated statistically significant reductions in symptoms from baseline to end of treatment in all six assessments measured. CY-BOCS results demonstrated statistically significant reductions in obsessive compulsive symptoms (*p* < 0.0001), resulting in >50% improvement sustained for at least 8 weeks after the final infusion and up to 46 weeks in a subset of subjects.

***Conclusions:*** In PANS, which may be associated with an underlying immune dysregulation, sequential infusions of IVIG [Octagam 5%] successfully ameliorated psychological symptoms and dysfunction, with sustained benefits for at least 8 weeks, and up to 46 weeks in a subset of subjects. In addition, baseline immune and autoimmune profiles demonstrated significant elevations in a majority of subjects, which requires further evaluation, characterization, and study to clarify the potential immune dysfunction by which PANS manifests and progresses.

## Introduction

Rapid onset of obsessive compulsive disorder (OCD) and/or tic disorder in children following streptococcal infections was initially explored at the National Institutes of Mental Health (NIMH) in the late 1990s. The researchers who initially reported this syndrome used the terminology, “pediatric autoimmune neuropsychiatric disorders associated with streptococcal infections,” or PANDAS, to describe the disorder (Swedo et al. 1998). The criteria established by the NIMH for the diagnosis of PANDAS included “(1) the presence of OCD and/or a tic disorder; (2) pediatric onset; (3) an episodic course of symptom severity; (4) an association with streptococcal infections; (5) an association with neurological abnormalities, including piano-playing choreiform movements of the fingers and toes, which suggests that PANDAS may be similar to Sydenham's chorea (SC)” (Swedo et al. 1998).

As a result of the inherent difficulties in defining the inciting incident/infection associated with PANDAS in a pediatric population, as well as a lack of precise testing modalities and biological markers, the diagnostic criteria were revised. A broader definition of this clinical entity was proposed. The preferred terminology is now, “pediatric acute-onset neuropsychiatric syndrome,” or PANS, in which the key clinical features include, “acute and dramatic symptom onset of OCD and/or severely restrictive food intake with at least two coinciding abrupt onset, equally debilitating symptoms (anxiety; dysregulation; irritability, aggression, oppositionality; behavioral regression; cognitive deterioration; sensory or motor abnormalities; somatic symptoms)” (Swedo et al. 2012). Based on these new criteria, PANDAS is now considered a subgroup of PANS.

The first PANS Consensus Conference was assembled at Stanford University in 2013 with a group of clinicians and researchers from several different geographic areas and specialties, including general and developmental pediatrics, infectious diseases, immunology, rheumatology, neurology, and child psychiatry. Because the diagnostic boundaries of PANS were ambiguous and somewhat debatable, the goal of the meeting was to develop key clinical and behavioral criteria. The result of this important conference was a Consensus Statement proposing recommendations for the diagnostic evaluation of youth presenting with PANS (Chang et al. 2015).

Guidelines for treating PANS/PANDAS were published as a three-part series of articles published in 2017 (Cooperstock et al. 2017; Frankovich et al. 2017; Thienemann et al. 2017) by the PANS Research Consortium (PRC). Current treatment modalities for PANS include psychiatric and behavioral interventions as well as the use of nonsteroidal anti-inflammatory drugs (NSAIDs), antibiotic therapy, corticosteroids, plasmapheresis, and intravenous immunoglobulin (IVIG). As per the guidelines, for moderate to severe PANS, oral or intravenous corticosteroids may be sufficient, however, IVIG is often the preferred treatment for these patients by most PRC members (Frankovich et al. 2017).

An increasing body of clinical, preclinical, and basic science research data support conceptualizing PANS and PANDAS as immune-mediated neurological disorders, similar to SC, and suggest that immune dysfunction may contribute to disease manifestation and progression (Hornig 2013; Hornig and Lipkin 2013; Frankovich et al. 2015; Murphy et al. 2015; Cutforth et al. 2016). The hypothesis is that PANS may represent a new form of postinfectious autoimmunity, through molecular mimicry, suggesting a potential mechanism by which the disorder evolves. Therefore, a multisite study was proposed to explore the efficacy of multiple, consecutive infusions of IVIG for PANS treatment.

## Methods

### Participants and study design

This open label study was conducted at three clinical/research sites in the United States: IMMUNOe Research Center (Centennial, CO); Midlands Pediatrics (Papillion, NE); and Allergy, Asthma & Immunology Relief Research Institute (Charlotte, NC). A central Institutional Review Board approved the study (IntegReview). Participants were recruited from direct referrals from clinicians as well as ClinicalTrials.gov (NCT03348618). The parents of participants provided informed consent, and study participants provided assent, when appropriate.

To be eligible for the study, participants between 4 and 16 years of age were required to have a diagnosis of moderate to severe PANS based on accepted criteria (Swedo et al. 2012) as validated by the Pediatric Acute Neuropsychiatric Symptom Scale (PANS Scale), Parent Version conducted during a prescreening phone call (for additional information, see Behavioral Assessments section) (PANS Scale 2012) (Supplementary Appendix S1). It is also important to note that all patients presented with symptoms that were not controlled using standard PANS therapy (e.g., antibiotics, selective serotonin reuptake inhibitors [SSRIs], corticosteroids, medications for attention-deficit/hyperactivity disorder [ADHD] such as methylphenidate, and so on). Therefore, according to published treatment recommendations, they required more aggressive immunomodulatory interventions (e.g., IVIG) (Frankovich et al. 2017). Antecedent therapies following date of onset of PANS symptoms (before enrollment), as well as PANS triggers, were reported during initial intake.

Participants who were using prophylactic antibiotics were required to be on a stable dose for ≥3 months. In addition, potential participants were excluded if they had a history of rheumatic fever, including SC (with neurologic manifestations), previous IVIG therapy within 6 months before screening, and/or use of corticosteroids within 6 weeks before screening. If potential participants had been prescribed antibiotics for an acute infection, a washout period of 7 days following completion of dose was required.

### Behavioral assessments

For the primary outcome measures, licensed independent (from the clinician's study center) psychologists administered validated psychometric scales, including the Children's Yale-Brown Obsessive Compulsive Scale (CY-BOCS), Clinical Global Impression of Severity (CGI-S), Yale Global Tic Severity Scale (YGTSS), and the Anxiety Disorders Interview Schedule for DSM-IV, Child/Parent versions (ADIS). In addition to these assessments, two parent-rated questionnaires were utilized during the study. The PANS Scale, Parent Version (PANS Scale 2012) (Supplementary Appendix S1) was administered as a prescreening measure for validation of the PANS diagnosis and to provide a baseline measurement of disease severity. Subsequent evaluations of the PANS Scale, following IVIG treatment, were also utilized to assess efficacy.

In addition to the PANS Scale, the Parent-Rated PANS Questionnaire (PRPQ) was developed specifically for this study and completed by parents at every treatment visit (Supplementary Appendix S2). This questionnaire takes 10–20 minutes to complete and contains 58 items selected as key symptoms of interest for data analysis per the most important PANS characteristics reported in the literature (Bernstein et al. 2010; Swedo et al. 2012).

### Exploratory assessments

Exploratory outcome measures included evaluation of key neuroimmune panels (Cunningham Panel [Moleculera Labs, Oklahoma City, OK], Neural Zoomer [Vibrant Wellness, San Carlos, CA]), as well as immune, infectious, and atopic laboratory panels. The Cunningham Panel includes five assays, including immunoglobulin G (IgG) levels by enzyme-linked immunosorbent assays directed against (1) dopamine D1 receptor (D1R), (2) dopamine D2L receptor (D2LR), (3) lysoganglioside-GM1, and (4) tubulin. A fifth assay is a cell stimulation assay which measures the ability of a patient's serum IgG to stimulate calcium/calmodulin-dependent protein kinase II (CaMKII) activity in human neuronal cells (Shimasaki et al. 2020). The Neural Zoomer panel evaluates 16 neurological autoantibodies, including, among others, antitubulin IgM/IgG+IgA, antimyelin basic protein IgM/IgG+IgA, antineuron-specific enolase IgM/IgG+IgA, and anti-GM1/GM2 IgM/IgG+IgA (Vibrant Wellness 2020).

Based on the work by Swedo et al. (2012), motor abnormalities occurring in PANS include a variety of signs and symptoms. Dysgraphia and fine motor skills may abruptly deteriorate following onset of symptoms. Therefore, obtaining a drawing sample during the acute phase, and during an asymptomatic period, is a relatively simple way to document motor changes. For these reasons, optional drawing/writing samples were collected from participants as an additional measure of assessment both before and following treatment.

### Safety assessments

All subjects were given a patient diary and asked to catalog all adverse events (AEs). In addition, a follow-up phone call 72 hours postinfusion by a research coordinator was also implemented to gather AEs. The parents were instructed to record the following data in the diary: any suspected AEs, temperature (using same method for every time), infections (serious acute bacterial infections had to be validated), physician/emergency room visits, hospitalizations (overnight stays), school/work days missed because of infections or illness, and concomitant medications, especially antibiotics. The diary was reviewed, and AEs were monitored, at every treatment visit following the first IVIG infusion.

### Visit schedule and procedures

The study consisted of a prescreening phone call, followed by 10 visits. During the prescreening phone call, the PANS Scale (2012) (Supplementary Appendix S1) was administered to assess disease severity. If the potential participant met the criteria of moderate to severe PANS, a subsequent on-site screening/baseline visit (Visit 0) was scheduled and included both the potential participant and parent(s). At Visit 0, medical history and concomitant/antecedent medications were assessed, baseline psychometric evaluations were conducted (CY-BOCS, CGI-S, YGTSS, ADIS), and blood was drawn for initial panel, biomarker, and safety assessments. In addition, optional pretreatment writing and/or drawing samples were gathered from participants and parents. Four weeks later, eligible participants received IVIG infusions every 21 days (± 3 days) for a total of 6 infusions over a period of 18 weeks (Visits 1–6). In addition to IVIG infusions, AEs (including review of diaries) and concomitant medications were assessed, and parents completed the PRPQ, at each treatment visit.

Follow-up included a visit ∼1 week after the final infusion (Visit 7) and a visit 7 weeks after the final infusion (Visit 8), the latter of which was considered the end of study (EOS) visit. At Visits 7 and 8, all psychometric evaluations (CY-BOCS, CGI-S, YGTSS, and ADIS) and the PANS Scale were administered. In addition, blood was drawn for posttreatment evaluation of all panel, biomarker, and safety assessments.

A late study visit (up to 46 weeks following the final infusion) was added to the study design to gather additional psychometric evaluations (CY-BOCS, CGI-S, YGTSS, and ADIS) in a subset of available participants (Visit 9) to assess durability of response.

### Study drug and dosage/administration

IVIG has been used to treat primary and secondary immunodeficiencies at replacement doses of 0.2–0.6 g/kg body weight every 3–4 weeks and enhances immune homeostasis by modulating expression and function of Fc receptors, interfering with activation of complement and production of cytokines, providing anti-idiotypic antibodies, and affecting the activation and effector functions of T and B cells (Cunningham-Rundles et al. 1984; Perez et al. 2017; Melamed et al. 2019). In higher doses of 1–2 g/kg body weight, IVIG has been shown to induce immune modulation and suppress systemic inflammation, and has long been used in the treatment of autoimmune and inflammatory conditions (Dwyer 1992; Nimmerjahn and Ravetch 2007; Ballow 2014; Joao et al. 2018).

The design of the study included on-site administration of IVIG [Octagam 5%] at a dosage of 1 g/kg of body weight every 21 days (±3 days) for a total of six infusions (cycles) over a period of 18 weeks. While a dose of 2 g/kg of IVIG has been used for immunomodulation in adults, we have found that it is a very large dose for pediatric patients and requires administration over 2–4 days. A dose of 1 g/kg can be administered in 1–2 days in the majority of pediatric patients, which is much more manageable in this population (Melamed et al. 2018). In our clinical experience before initiation of this study, we also found a dose of 1 g/kg to be effective in reducing/eliminating symptoms in PANS patients. In a previous study in pediatric patients with autism spectrum disorder (ASD), a dose of 1 g/kg of a 5% IVIG was well tolerated and significant improvements in behavioral and cognitive assessments were demonstrated (Melamed et al. 2018). A dose of 1 g/kg has also been shown to be effective in pediatric patients with immune thrombocytopenic purpura (Warrier et al. 1997).

The IVIG study drug [Octagam 5%] was specifically chosen based on our positive clinical experiences of tolerability in pediatric patients (Melamed et al. 2018). Although higher percentage concentrations are available, we prefer 5% (vs. 10%), again, due to our perception of improved clinical tolerability in this population. The study drug was provided in bottles from the manufacturer [Octapharma], and was labeled and stored appropriately for investigational use. The study drug was administered intravenously directly from the bottle by a health care provider according to the labeled infusion rates (which should not exceed 3.33 mg/kg/min [200 mg/(kg·h)]. Vital signs were monitored throughout each infusion.

It is important to note that the number of sequential IVIG infusion cycles ( × 6) evaluated in this study is a unique treatment model that, to the best of our knowledge, has not been utilized in any previously reported assessment of IVIG treatment efficacy in the PANS population.

### Statistical analysis

Unadjusted descriptive statistics were conducted to summarize the endpoints for eligible participants to detect the mean, standard deviation for continuous variables, and percentages for categorical variables. In adjusted descriptive statistics, outliers present in data sets will often be removed to determine the adjusted mean because they can have a large impact on the calculated means of small populations. To maintain the integrity of the data, we did not adjust the statistics in this manner to correct statistical averages to compensate for data imbalances and variances. Differences between subjects were tested using Student's *t* test for continuous variables and Fisher's exact tests were used for categorical variables. Analyses were conducted using SAS 9.4 software (SAS Institute, Cary, NC). A two-sided *p*-value <0.05 was considered statistically significant.

## Results

### Study population

A total of 26 patients were screened and 21 patients met the criteria for participation in the study (7 subjects at each site) (Table 1). The five screened patients who were unable to participate had scheduling conflicts related to IVIG infusion dates, decided that they did not want to participate, or did not meet inclusion criteria for severity. The enrolled subjects included 13 males (62%) and 8 females (38%). The majority of subjects were white with a mean age of 10.86 ± 2.88 and weight of 43.83 kg ±21.88. The onset of PANS symptoms was obtained for all subjects enrolled in the study. The mean number of years of PANS symptoms before enrollment was 4.3 ± 2.2 (range of 3–9 years). There were 14 subjects (67%) who had a streptococcal infection associated with their initial onset of PANS symptoms. In the remaining subjects, PANS was associated with sinusitis and/or respiratory infection (*n* = 3 [14%]) or the etiology was unknown (*n* = 4 [19%]). Antecedent medications for PANS were also recorded at enrollment. All subjects (*n* = 21 [100%]) had received antibiotics. There were three subjects (14%) who had received corticosteroids, three subjects (14%) who had received SSRIs, and three subjects (14%) who had received ADHD medications (methylphenidate, guanfacine). In addition, one subject had previously received two infusions of IVIG ∼6 years before study start. Several subjects (*n* = 12 [57%]) had also received NSAIDs and/or antihistamines as needed for symptom management. However, because these medications are available over-the counter, it is difficult to accurately quantify use before enrollment.

**Table 1.** Sociodemographic and Baseline Clinical Characteristics

Characteristic	*n* (%)	Mean ± SD
Age	21 (100)	10.86 ± 2.88
Sex		
Male	13 (62)	
Female	8 (38)	
Race		
White	19 (90)	
Asian	1 (5.0)	
Asian/White	1 (5.0)	
Weight (kg)	20 (95)	43.83 ± 21.18
PANS with streptococcal relationship	14 (67)	
Years of PANS symptoms (before enrollment)		4.3 ± 2.2
PANS Scale, OCD Symptom Score (0–25)	19 (90)	21.32 ± 5.22
CY-BOCS total (0–40)	21 (100)	22.10 ± 7.82
CGI-S		4.67 ± 0.84
Moderate (4)	10 (48)	
Marked (5)	6 (28)	
Severe (6)	5 (24)	
CaMKII		
Serum	21 (100)	130.85 ± 25.01
Elevated (>130)	7 (33)	
Antitubulin antibodies		
Serum	21 (100)	1880.95 ± 1252.66
Elevated (≥1000)	20 (95)	

CaMKII, calcium/calmodulin-dependent protein kinase II; CGI-S, Clinical Global Impressions of Severity; CY-BOCS, Children's Yale-Brown Obsessive Compulsive Scale; OCD, obsessive compulsive disorder; PANS Scale, Pediatric Acute Neuropsychiatric Symptoms Scale; SD, standard deviation.

As expected, the mean PANS Scale OCD Symptom Score at baseline was high at 21.32 ± 5.22 (scoring system of 0–25). As per the CGI-S, 10 (48%) of participants presented with moderate PANS symptoms, 6 (28%) with marked symptoms, and 5 (24%) were considered severe. Again, it should be noted that mean baseline serum measurements of CaMKII and antitubulin antibodies were both elevated.

### Primary efficacy endpoints

**FIG. 1.**
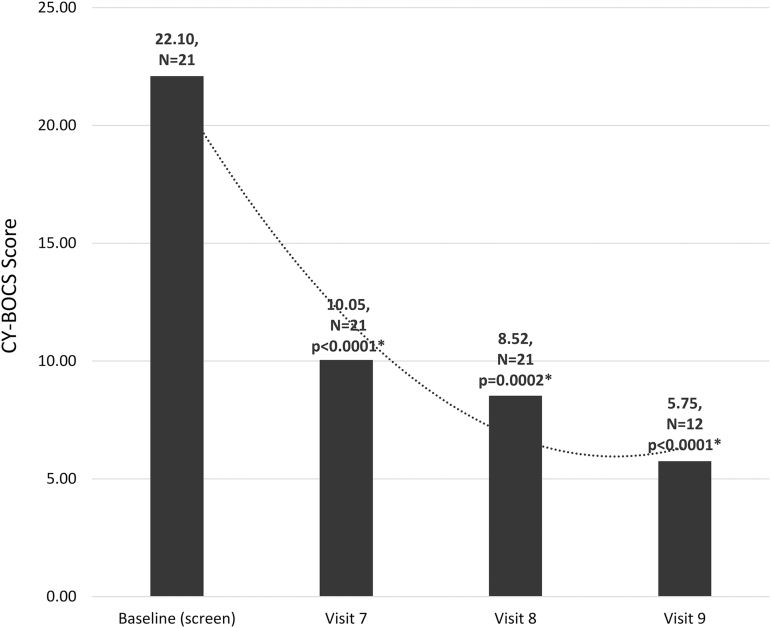
Unadjusted mean CY-BOCS total scores (*p < 0.05 was considered statistically significant). Note that in a subset of subjects (*n* = 12) who participated in a late follow-up visit (29–46 weeks following the final infusion), results continued to improve compared to baseline. The timing of evaluations is as follows: Visit 7 (19 weeks after baseline), Visit 8 (26 weeks after baseline), Visit 9 (29–46 weeks after Visit 8/final infusion and 55–72 weeks after baseline). CY-BOCS, Children's Yale-Brown Obsessive Compulsive Scale.

**FIG. 2.**
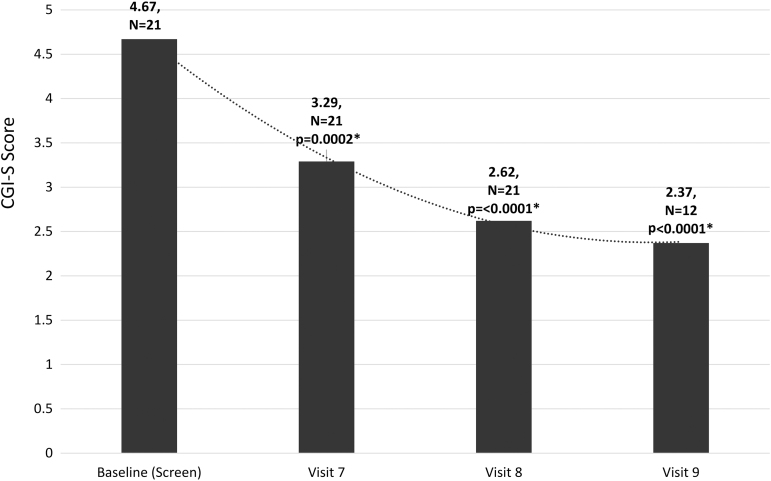
Unadjusted mean CGI-S scores (*p < 0.05 was considered statistically significant). Note that in a subset of subjects (*n* = 12) who participated in a late follow-up visit (29–46 weeks following the final infusion), results continued to improve compared to baseline. The timing of evaluations is as follows: Visit 7 (19 weeks after baseline), Visit 8 (26 weeks after baseline), Visit 9 (29–46 weeks after Visit 8/final infusion and 55–72 weeks after baseline). CGI-S, Clinical Global Impression of Severity.

**FIG. 3.**
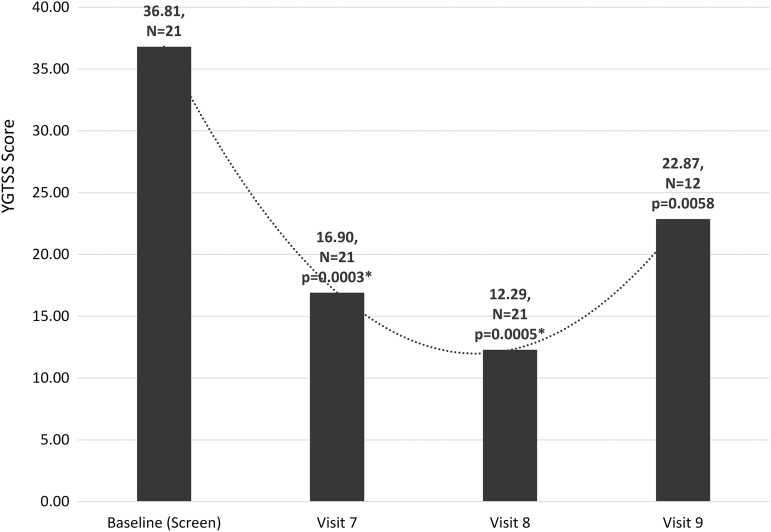
Unadjusted mean YGTSS scores (*p < 0.05 was considered statistically significant). Note that in a subset of subjects (*n* = 12) who participated in a late follow-up visit (29–46 weeks following the final infusion), results indicate that tics returned, although they were still below baseline levels. The timing of evaluations is as follows: Visit 7 (19 weeks after baseline), Visit 8 (26 weeks after baseline), and Visit 9 (29–46 weeks after Visit 8/final infusion and 55–72 weeks after baseline). YGTSS, Yale Global Tic Severity Scale.

**FIG. 4.**
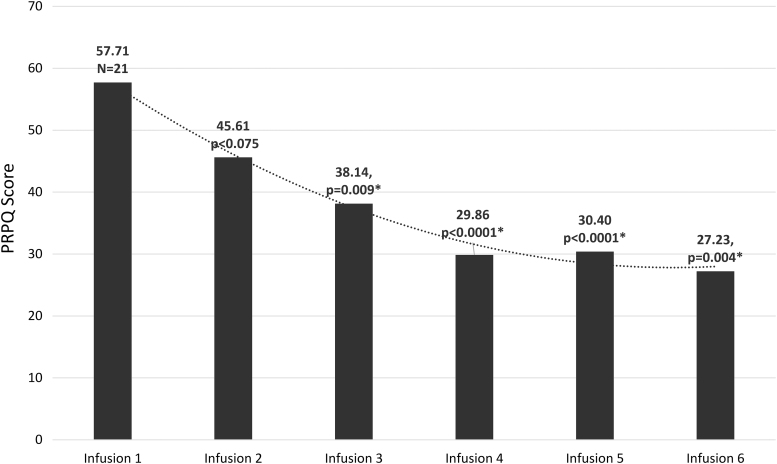
Unadjusted mean scores from infusion 1 to infusion 6 (infusions occurred every 3 weeks) of the PRPQ (Supplementary Appendix S2) (*p < 0.05 was considered statistically significant). This questionnaire takes 10–20 minutes to complete and contains 58 items selected as key symptoms of interest for data analysis per the most important PANS characteristics reported in the literature. The importance of this assessment, compared to the others conducted in this study, is that it demonstrates the efficacy of IVIG following each infusion. Statistically significant reductions in symptoms were noted by the third IVIG infusion. IVIG, intravenous immunoglobulin; PANS, pediatric acute-onset neuropsychiatric syndrome; PRPQ, Parent-Rated Pediatric Acute-Onset Neuropsychiatric Syndrome Questionnaire.

The primary efficacy endpoints were validated psychometric assessments (CY-BOCS, CGI-S, YGTSS, and ADIS) and parent observations (PANS Scale, PRPQ). Statistically significant improvements were demonstrated in all psychometric assessments and parent questionnaires from baseline to end of treatment and in early/late follow-up visits (Figs. 1–4 and Tables 2 and 3). In a subset of subjects (*n* = 12) who participated in a late follow-up visit (29–46 weeks following the final infusion), results indicate that tics returned, although they were still below baseline levels (Fig. 3 and Table 2). One of the most important assessments was the PRPQ, in that it demonstrates the efficacy of IVIG following each infusion (Fig. 4 and Table 3). As the other primary efficacy assessments were only performed at baseline, the final infusion (Visit 7), and early/late follow-up visits (Visits 8 and 9), the PRPQ is the only assessment that provides interim efficacy data. Statistically significant reductions in symptoms were noted by the third IVIG infusion per the PRPQ assessment data (Fig. 4 and Table 3).

**Table 2.** Behavioral Assessment Outcomes

Assessment	Baseline (screen)	Visit 7	Visit 8	Visit 9^a^
CY-BOCS mean total scores ± SD	22.10 ± 8.02	10.05 ± 8.53	8.52 ± 9.70	5.75 ± 8.53
Percentage change^b^		−54.52%	−61.45%	−71.01%
Mean change^b^		−12.05	−13.58	−14.08
CY-BOCS *p* values^c^		<0.0001	0.0002	<0.0001
CGI-S mean scores ± SD	4.67 ± 0.86	3.29 ± 1.35	2.62 ± 1.13	2.37 ± 1.30
Percentage change^b^		−29.55%	−43.90%	−46.23%
Mean change^b^		−1.38	−2.05	−2.04
CGI-S *p* values^c^		0.0002	<0.0001	<0.0001
YGTSS mean scores ± SD	36.81 ± 26.67	16.90 ± 19.16	12.29 ± 14.27	22.87 ± 26.46
Percentage change^b^		−54.09%	−66.61%	−44.70%
Mean change^b^		−19.91	−24.52	−18.50
YGTSS *p* values^c^		0.0003	0.0005	0.0058

^a^ Subset of patients (*n* = 12) that participated in a late follow-up visit 29–46 weeks after Visit 8/final infusion and 55–72 weeks after baseline (screen). Percentage change and mean change were only calculated for the subset of participating patients.

^b^ Percentage and mean change calculated from baseline (screen).

^c^ *p*-Values <0.05 were considered statistically significant.

CGI, Clinical Global Impressions of Severity; CY-BOCS, Children's Yale-Brown Obsessive Compulsive Scale; SD, standard deviation; YGTSS, Yale Global Tic Severity Scale.

**Table 3.** Parent-Rated Pediatric Acute-Onset Neuropsychiatric Syndrome Questionnaire Outcomes

PRPQ	Infusion 1	Infusion 2	Infusion 3	Infusion 4	Infusion 5	Infusion 6
Mean scores ± SD	57.71 ± 36.40	45.61 ± 31.21	38.14 ± 24.77	29.86 ± 22.17	30.40 ± 26.25	27.23 ± 33.23
Percentage change^a^		20.97%	34.22%	48.26%	47.32%	52.82%
Mean change^a^		−12.10	−19.75	−27.85	−27.31	−30.48
PRPQ *p* values^b^		0.075	0.009	<0.0001	<0.0001	0.004

^a^ Percentage and mean change calculated from infusion 1.

^b^ *p*-Values <0.05 were considered statistically significant.

PRPQ, Parent-Rated PANS Questionnaire; SD, standard deviation.

### Exploratory endpoints

#### Biomarker evaluations

Several baseline immune, atopic, and infectious laboratory variables as well as neuroimmune panels (Cunningham Panel, Neural Zoomer) were explored as possible predictors or moderators of response. Of these, only CaMKII elevation (*n* = 7) (Table 1) was found to be potentially related to response based on CY-BOCS total scores at EOS. While there was a minor difference in mean CY-BOCS total score between the two groups (elevated CaMKII CY-BOCS score: 10.5 ± 10.7; normal CaMKII CY-BOCS score: 7.4 ± 8.4), the difference did not reach statistical significance.

#### Drawing/writing samples

**FIG. 5.**
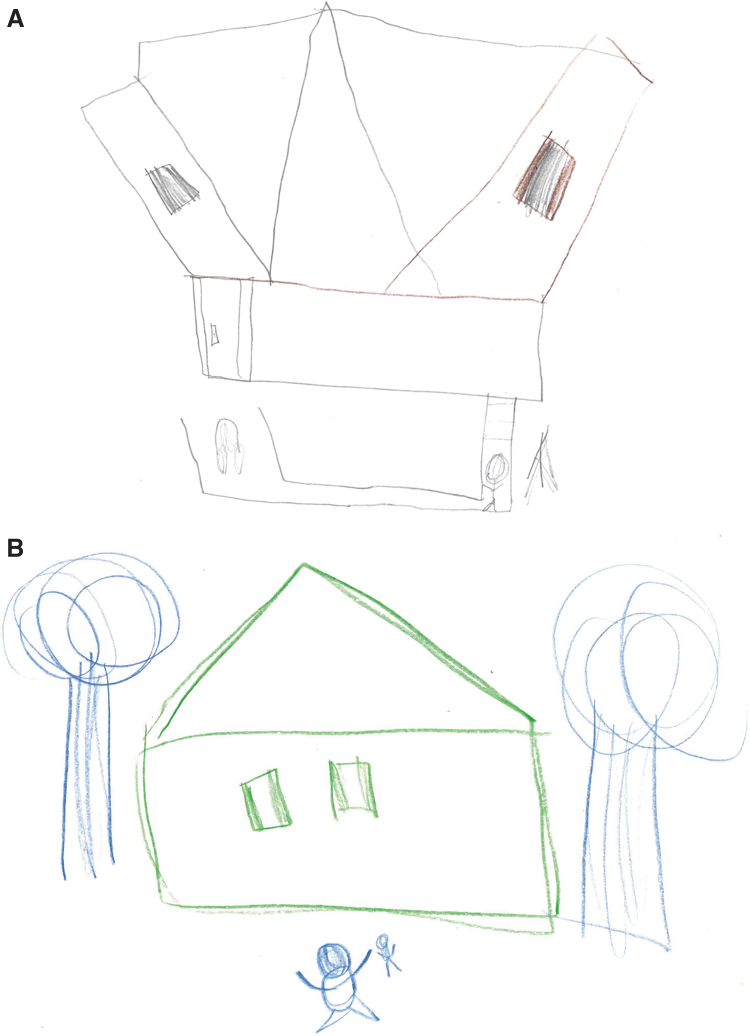
The subject was asked to draw a house. **(A)** Subject's drawing before treatment. **(B)** Subject's drawing following IVIG treatment. IVIG, intravenous immunoglobulin.

**FIG. 6.**
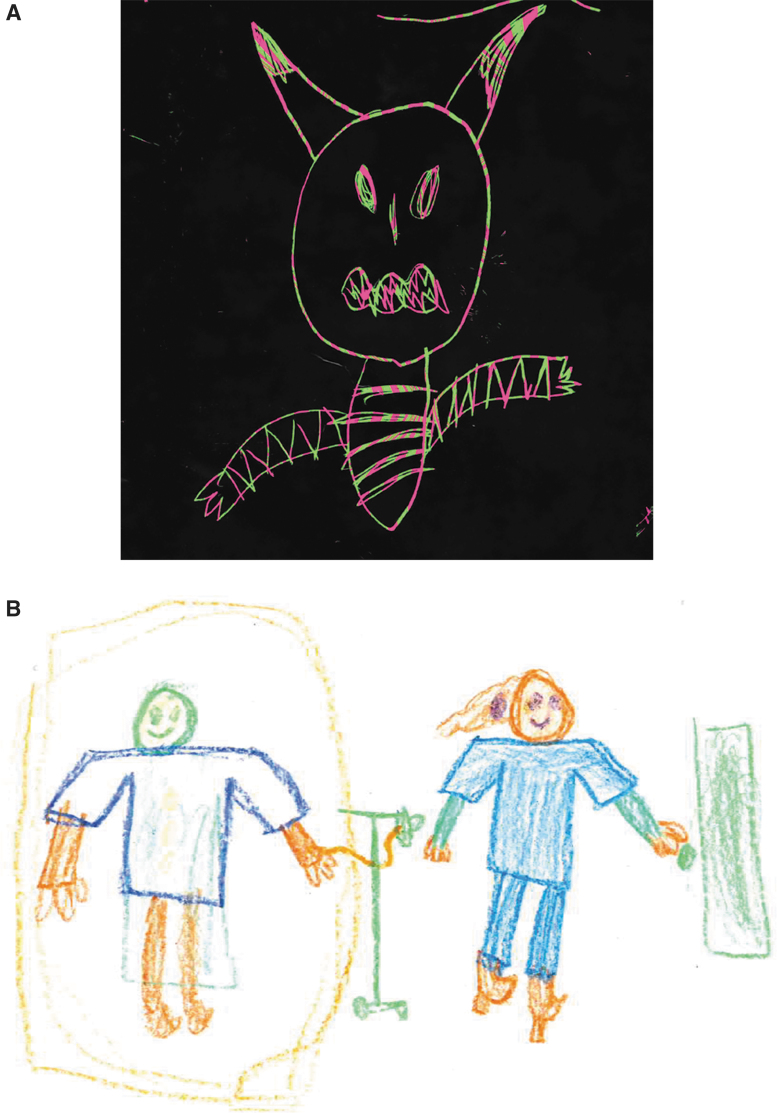
The subject was asked to draw, “self and others.” **(A)** Subject's drawing before treatment. **(B)** Subject's drawing following IVIG treatment. IVIG, intravenous immunoglobulin.

A dramatic example of the potent effects of IVIG in this patient population is demonstrated in drawing samples of the PANS subjects before and after the administration of IVIG (Figs. 5 and 6). As can be seen in the examples, drawing skills/perspective and mood/outlook may abruptly deteriorate following onset of symptoms with resolution following immunomodulatory treatment.

### Adverse events

AEs that were considered related to the IVIG infusions included three severe headaches. One patient required sumatriptan, and no medication was required for resolution in the other two subjects. Following resolution, there were no further complications.

The remaining related AEs were rated as mild or moderate and included headache, (*n* = 10 [48%]), nausea/vomiting (*n* = 3 [14%]), and rash (*n* = 3[14%]). All mild and moderate AEs resolved without further complication. No serious AEs occurred during the study.

## Discussion

To the best of our knowledge, this is the first study to assess a total of six infusions for the treatment of PANS. The results of this prospective, open-label, proof-of-concept study substantiate earlier randomized, controlled clinical trials of the benefits of IVIG in controlling PANS symptoms (Perlmutter et al. 1999; Williams et al. 2016), however, the extended dosing strategy in this study demonstrated durability of effects up to 46 weeks following the final infusion. It is notable that per the interim measurements provided by the PRPQ, statistically significant drops in symptom scores did not occur until third infusion (Fig. 4 and Table 3). The dosing strategy in earlier randomized, controlled studies was 1 g/kg administered over 2 consecutive days (2 g/kg total) (Perlmutter et al. 1999; Williams et al. 2016). In this study, we utilized a total dose of 1 g/kg every 3 weeks for a total of six infusions. While a dose of 2 g/kg of IVIG is routinely used for immunomodulation in adults, it is a very large dose in the pediatric population and must be administered over 2–4 days. A dose of 1 g/kg can be administered in 1–2 days in the majority of pediatric patients, which is much more manageable in this population (Melamed et al. 2018). In a previous study in pediatric patients with ASD conducted by the lead author, a dose of 1 g/kg of a 5% IVIG was well tolerated. In addition, significant improvements in behavioral and cognitive assessments were demonstrated (Melamed et al. 2018). A total dose of 1 g/kg of IVIG has also been shown to be effective in pediatric patients with immune thrombocytopenic purpura (Warrier et al. 1997). The study drug [Octagam 5%] was specifically chosen based on our positive clinical experiences of tolerability in pediatric patients, which was also demonstrated in this study and the previous study in ASD (Melamed et al. 2018).

In the first double-blind, placebo-controlled investigation conducted by Perlmutter et al. (1999), therapeutic plasma exchange (TPE; five single-volume exchanges over 2 weeks), IVIG (1 g/kg daily on 2 consecutive days), or placebo (saline solution given in the same manner as IVIG) were compared. Results demonstrated that IVIG and TPE were both effective in reducing OCD symptoms in PANDAS patients (by 45% and 58%, respectively), whereas a placebo infusion had no discernable effect (Perlmutter et al. 1999). In contrast, non-PANDAS OCD (Nicolson et al. 2000) and tic disorders (Hoekstra et al. 2004) do not demonstrate benefits in TPE and IVIG, respectively.

Although the use of IVIG in the treatment of PANS has been utilized clinically, no additional placebo-controlled trials were conducted until 2016 (Williams et al. 2016). The study consisted of four visits: baseline, week 6 (end of the blinded phase), week 12 (end of the open-label phase), and week 24 (follow-up). At baseline, participants received either IVIG (2 g/kg per day administered at 1 g/kg over 2 days; *n* = 17) or placebo (*n* = 18). Six weeks following baseline, participants were evaluated, and a “responder” was defined as a decrease in CY-BOCS score of ≥30%, and “Much” or “Very Much” improved rating on CGI-I. Nonresponders to the blinded infusion were offered an open-label IVIG infusion.

At 6 weeks, the mean decrease in OCD severity was greater in the IVIG cohort than in placebo, but this difference did not reach statistical significance. It was determined that the study's power to detect between-group differences was tempered by the high variability in individual improvement after double-blind administration of IVIG. It was also known to the participants and their parents that those who did not meet the criteria for a “responder” in the 6-week portion of the study would receive an open-label IVIG infusion. The OCD severity scores for those receiving open-label IVIG (regardless of whether they had received a placebo or blinded IVIG infusion) decreased roughly 50% in 6 weeks. Because these improvements were only demonstrated during the open-label phase of the trial, it was not possible to definitively determine the efficacy of IVIG. In particular, participants may have overreported symptom severity in the double-blind portion of the study to increase the possibility of getting open-label IVIG at 6 weeks.

The limitations of this study include the small sample size, lack of a control group, and a heterogeneous patient population with differing durations of illness and antecedent treatments, as well as diverse PANS triggers (although the majority did have a relationship to a streptococcal infection). Although neuroimmune, atopic, and biomarker panels from baseline to end of treatment were explored, interpretation of results was complicated by the interference of the immunologic components of IVIG following infusion. The clinical laboratory testing problems associated with IVIG have been reported (Branch 2019), including interference with accurate antibody detection and antiglobulin testing. In future studies, it may be useful to include late neuroimmune, atopic, and biomarker panels in the design (e.g., following a washout period of 6–12 months) for comparison to baseline assessments. In addition, it is important to note the inherent difficulties in measuring systemic serum biomarkers for a localized brain disease such as PANS. It may be that the immunologic “action” is localized within brain tissue and central nervous system, and blood measurements are too remote, diffuse, and insensitive. In animal models that include cerebrospinal fluid measurements, brain tissue biopsies, and so on, results are impressive and convincing. Obviously, such studies are difficult, if not impossible, to conduct in children for a variety of reasons.

The positive results from this study contribute to the gathering evidence in support of conceptualizing PANS as an immune-mediated brain disease, similar to SC, involving the caudate, putamen, and other basal ganglia structures. Published data support the premise that PANS is an autoimmune disorder in susceptible children resulting in immune dysregulation involving autoantibodies, autoreactive T cells, disruption in T-regulatory cell function, microglial cell dysregulation, inappropriate release of or response to inflammatory cytokines, and autoreactive B cells, which result in an inflammatory disorder of the basal ganglia (Hornig 2013; Hornig and Lipkin 2013; Williams and Swedo 2015; Cutforth et al. 2016; Frick and Pittenger 2016; Frankovitch et al. 2017). Therefore, the use of a broad-spectrum immunomodulatory agent, such as IVIG, should result in changes in behavior brought on by abnormal inflammation (Ballow 2014; Spinello et al. 2016; Frankovitch et al. 2017; Joao et al. 2018). In other words, if PANS were not an autoimmune, autoinflammatory disease, then an immunomodulatory intervention, such as IVIG, should not have any impact on psychometric and clinical measurements. As the results of our study demonstrate, sequential infusions of IVIG had a significant, positive impact on PANS patients, supporting the characterization of PANS as an autoimmune disorder.

## Conclusions

The results of this study demonstrated that in PANS, which may be associated with an underlying immune dysregulation, sequential infusions of IVIG [Octagam 5%] successfully ameliorated psychological symptoms and dysfunction, with sustained benefits for at least 8 weeks, and up to 46 weeks in a subset of subjects, following the final infusion. In addition, baseline immune and autoimmune profiles demonstrated significant elevations in a majority of subjects, which requires further evaluation, characterization, and study to clarify the potential immune dysfunction by which PANS manifests and progresses.

## Clinical Significance

The limitations of this open-label pilot study include the small sample size and lack of a control group. However, in this population of PANS subjects, all psychometric endpoints studied exhibited statistically significant decreases following six infusions of IVIG. These positive results warrant a randomized, placebo-controlled trial to definitively evaluate the impact of multiple, sequential IVIG infusions on PANS symptoms. The durability of response is also noteworthy. Although the majority of PANS symptoms were still under control at the late follow-up visit (up to 46 weeks), it is of interest that tics returned in a subset of subjects following washout of IVIG. For these patients, additional infusions may be required to ameliorate recurrent symptoms.

## Disclosures

I.M. has received honoraria and research support from Octapharma AG; he has also received honoraria and research support from the Pharming group. R.H.K. has received honoraria and research support from Octapharma AG, research support and honoraria from Takeda (previously Baxalta/Shire), research support from the Vietnam Respiratory Society, Hanoi Vietnam, research support from Vietnam National Children and Hospital Hanoi, Vietnam, and personal fees/honoraria from UCLA School of Medicine. M.O. has received honoraria and research support from Octapharma AG. A.L.K. has received honoraria, research support from Octapharma AG. All other authors have nothing to disclose other than their employment affiliations.

## Supplementary Material

Supplemental data

Supplemental data

## References

[B1] Ballow M: Mechanisms of immune regulation by IVIG. Curr Opin Allergy Clin Immunol 14:509–515, 2014 25337683 10.1097/ACI.0000000000000116

[B2] Bernstein GA, Victor AM, Pipal AJ, William KA: Comparison of clinical characteristics of pediatric acute autoimmune neuropsychiatric disorders associated with streptococcal infections and childhood obsessive-compulsive disorder. J Child Adolesc Psychopharmacol 20:333–340, 2010 20807071 10.1089/cap.2010.0034 PMC3678581

[B3] Branch DR: Serologic problems associated with administration of intravenous immune globulin (IVIg). Immunohematology 35:13–14, 2019 30908073

[B4] Chang K, Frankovich J, Cooperstock M, Cunningham MW, Latimer ME, Murphy TK, Pasternack M, Thienemann M, Williams K, Walter J, Swedo SE: Clinical evaluation of youth with pediatric acute-onset neuropsychiatric syndrome (PANS): Recommendations from the 2013 PANS Consensus Conference. J Child Adolesc Psychopharmacol 25:3–13, 2015 25325534 10.1089/cap.2014.0084 PMC4340805

[B5] Cooperstock M, Swedo S, Pasternack M, Murphy T: Clinical management of pediatric acute-onset neuropsychiatric syndrome (PANS): Part III—Treatment and prevention of infections. J Child Adolesc Psychopharmacol 27:594–606, 2017 10.1089/cap.2016.0151 PMC9836684 36358106

[B6] Cunningham-Rundles C, Siegel FP, Smithwick EM, Lion-Boule A, Cunningham-Rundles S, O'Malley J, Barandun S, Good RA: Efficacy of intravenous immunoglobulin in primary humoral immunodeficiency disease. Ann Intern Med 101:435–439, 1984 6206756 10.7326/0003-4819-101-4-435

[B7] Cutforth T, DeMille MM, Agalliu I, Agalliu D: CNS autoimmune disease after infections: Animal models, cellular mechanisms and genetic factors. Future Neurol 11:63–76, 2016 27110222 10.2217/fnl.16.4 PMC4839655

[B8] Dwyer JM: Manipulating the immune system with immunoglobulin. N Engl J Med 326: 4104–4109, 1992 10.1056/NEJM199201093260206 1727218

[B9] Frankovich J, Swedo S, Murphy T, Dale RC, Agalliu D, Williams K, Daines M, Hornig M, Chugani H, Sanger T, Muscal E, Pasternack M, Cooperstock M, Gans H, Zhang Y, Cunningham M, Bernstein G, Bromberg R, Willet T, Brown K, Farhadian B, Chang K, Geller D, Hernandez J, Sherr J, Shaw R, Latimer E, Leckman J, Thienemann M: Clinical management of pediatric acute-onset neuropsychiatric syndrome (PANS): Part II—Use of immunomodulatory therapies. J Child Adolesc Psychopharmacol 27:574–593, 2017 10.1089/cap.2016.0148 PMC9836706 36358107

[B10] Frankovich J, Thienemann M, Pearlstein J, Crable A, Brown K, Chang K: Multidisciplinary clinic dedicated to treating youth with pediatric acute-onset neuropsychiatric syndrome: Presenting characteristics of the first 47 consecutive patients. J Child Adolesc Psychopharmacol 25:38–47, 2015 25695943 10.1089/cap.2014.0081 PMC4340335

[B11] Frick L, Pittenger C: Microglial dysregulation in OCD, Tourette syndrome, and PANDAS. J Immunol Res 2016:108, 2016 10.1155/2016/8606057 PMC5174185 28053994

[B12] Hoekstra PJ, Minderaa RB, Kallenberg CG: Lack of effect of intravenous immunoglobulins on tics: A double-blind placebo-controlled study. J Clin Psychiatry 65:537–542, 2004 15119917 10.4088/jcp.v65n0413

[B13] Hornig M: The role of microbes and autoimmunity in the pathogenesis of neuropsychiatric illness. Curr Opin Rheumatol 25:488–795, 2013 23656715 10.1097/BOR.0b013e32836208de

[B14] Hornig M, Lipkin WI: Immune-mediated animal models of Tourette syndrome. Neurosci Biobehav Rev 37:1120–1138, 2013 23313649 10.1016/j.neubiorev.2013.01.007 PMC4054816

[B15] Joao C, Negi VS, Kazatchkine MD, Bayry J, Kaveri SV: Passive serum therapy to immunomodulation by IVIG: A fascinating journey of antibodies. J Immunol 200;195 7–1963, 2018 10.4049/jimmunol.1701271 29507120

[B16] Melamed I, Heffron M, Dana R, Testori A, Rashid N: Observational study of intravenous immunoglobulin 5% for alleviating adverse drug reactions in primary immunodeficiency disorders. J Clin Cell Immunol 10:3, 2019

[B17] Melamed I, Heffron M, Testori A, Lipe K: A pilot study of high-dose intravenous immunoglobulin 5% for autism: Impact on autism spectrum and markers of neuroinflammation. Autism Res 11:421–433, 2018 29427532 10.1002/aur.1906

[B18] Murphy TK, Patel PD, McGuire JF, Kennel A, Mutch PJ, Parker-Athill EC, Hanks CE, Lewin AB, Storch EA, Toufexis MD, Dadlani GH, Rodriguez CA: Characterization of the pediatric acute-onset neuropsychiatric syndrome phenotype. J Child Adolesc Psychopharmacol 25:14–25, 2015 25314221 10.1089/cap.2014.0062 PMC4340632

[B19] Nicolson R, Swedo SE, Lenane M, Bedwell J, Wudarsky M, Gochman P, Hamburger SD, Rapoport JL: An open trial of plasma exchange in childhood-onset obsessive-compulsive disorder without poststreptococcal exacerbations. J Am Acad Child Adolesc Psychiatry 39:1313–1315, 2000 11026187 10.1097/00004583-200010000-00020

[B20] Nimmerjahn F, Ravetch JV: The antinflammatory activity of IgG: The intravenous IgG paradox. J Exp Med 204:11–15, 2007 17227911 10.1084/jem.20061788 PMC2118416

[B21] Pediatric acute neuropsychiatric symptoms scale, parent version (PANS Scale). 2012. Available at: http://pandasnetwork.org/wp-content/uploads/2018/11/pandas_pans_scale.pdf (accessed 220, 2020)

[B22] Perez EE, Orange JS, Bonila F, Chinen J, Chinn IK, Dorsey M, El-Gamal Y, Harville TO, Hossny E, Mazer B, Nelson R, Secord E, Jordan SC, Stiehm R, Vo AA, Ballow M: Update on the use of immunoglobulin in human disease: A review of evidence. J Allergy Clin Immunol 139:S1–S46, 2017 28041678 10.1016/j.jaci.2016.09.023

[B23] Perlmutter SJ, Leitman SF, Garvey MA, Hamburger S, Feldman E, Leonard HL, Swedo SE: Therapeutic plasma exchange and intravenous immunoglobulin for obsessive-compulsive disorder and tic disorders in childhood. Lancet 354:1153–1158, 1999 10513708 10.1016/S0140-6736(98)12297-3

[B24] Shimasaki C, Frye RE, Trifiletti R, Cooperstock M, Kaplan G, Melamed I, Greenberg R, Katz A, Fier E, Kem D, Traver D, Dempsey T, Latimer E, Cross A, Dunn JP, Bentley R, Alvarez K, Reim S, Appleman J: Evaluation of the Cunningham Panel^™^ in pediatric autoimmune neuropsychiatric disorder associated with streptococcal infection (PANDAS) and pediatric acute-onset neuropsychiatric syndrome (PANS): Changes in antineuronal antibody titers parallel changes in patients symptoms. J Neuroimmunol 339:577138, 2020 31884258 10.1016/j.jneuroim.2019.577138

[B25] Spinello C, Laviola G, Macri S: Pediatric autoimmune disorders associated with streptococcal infections and Tourette's syndrome in preclinical studies. Front Neurosci 10:310, 2016 27445678 10.3389/fnins.2016.00310 PMC4928151

[B26] Swedo SE, Leckman JF, Rose NR: Modifying the PANDAS criteria to describe PANS (pediatric acute-onset neuropsychiatric syndrome). Pediatr Ther 2:1–8, 2012

[B27] Swedo SE, Leonard HL, Garvey M, Mittleman B, Allen AJ, Perlmutter S, Lougee L, Dow S, Zamkoff J, Dubbert BK: Pediatric autoimmune neuropsychiatric disorders associated with streptococcal infections: Clinical description of the first 50 cases. Am J Psychiatry 155:264–271, 1998 9464208 10.1176/ajp.155.2.264

[B28] Thienemann M, Murphy T, Williams K, Leckman J, Shaw R, Geller D, Kapphahn C, Frankovich J, Elia J, Chang K, Hommer R, Swedo S: Clinical management of pediatric acute-onset neuropsychiatric syndrome (PANS): Part I—Psychiatric and behavioral interventions. J Child Adolesc Psychopharmacol 27:566–573, 2017 28722481 10.1089/cap.2016.0145 PMC5610394

[B29] Vibrant Wellness. Neural Zoomer. Available at: https://www.vibrant-wellness.com/tests/neural-zoomer/#1527504422745-6625ac95-ec67 (accessed 1218, 2020)

[B30] Warrier I, Bussel JB, Valdez L, Barbosa J, Beardsley DS: Safety and efficacy of low-dose intravenous immunoglobulin (IVIG) treatment for infants and children with immune thrombocytopenic purpura. J Pediatr Hematol Oncol 19:197–201, 1997 9201140 10.1097/00043426-199705000-00004

[B31] Williams KA, Swedo SE, Farmer CA, Grantz H, Grant PJ, D'Souza P, Hommer R, Katsovich L, King RA, Leckman JF: Randomized, controlled trial for pediatric autoimmune neuropsychiatric disorders associated with streptococcal infections. J Am Acad Child Adolesc Psychiatry 55:860–867, 2016 27663941 10.1016/j.jaac.2016.06.017

[B32] Williams KE, Swedo SE: Post-infectious autoimmune disorders: Sydenham's chorea, PANDAS and beyond. Brain Res 1617:144–154, 2015 25301689 10.1016/j.brainres.2014.09.071

