# Supplementary material for: Evaluation of Intravenous Immunoglobulin in Pediatric Acute-Onset Neuropsychiatric Syndrome
Source: J Child Adolesc Psychopharmacol. 2021 Mar 15;31(2):118–28. doi: 10.1089/cap.2020.0100 (PMC7984935; doi:10.1089/cap.2020.0100)
Supplement: Supplemental data [file Supp_App1.pdf]

PEDIATRIC ACUTE NEUROPSYCHIATRIC SYMPTOM SCALE* Parent version

Date: **Name:** **Gender:** **F** **M**

Date of birth:

Date of onset:

Informants: **Telephone numbers**

Version: June 6, 2012

**DOMAIN**	**One week prior to 1st Onset**	**Week following 1st Onset**	**Current (past 7 days)**
Date			
Obsessive-compulsive symptoms (0-25)<br>(5 X the worst of the OC symptoms)**			
Associated neuropsychiatric (NP) symptoms (0-25)<br>(sum of the 5 (of 7) worst NP domains)***			
1. Anxiety symptoms (0-5)			
2. Extreme moodiness and/or depression (0-5)			
3. Irritability or aggressive behavior (0-5)			
4. Learning/cognitive symptoms, confusion (0-5)			
5. Behavioral regression (0-5)			
6.A. Sensory symptoms (0-5)			
6.B. Hallucinations (0-5)			
6.C. Motor symptoms (0-5)			
7.A. Urinary symptoms (0-5)			
7.B. Sleep disturbance, fatigue (0-5)			
7.C. Dilated pupils (0-5)			
TOTAL SYMPTOMS (0-50)			
Impairment (0-50)			
TOTAL SCORE (0-100)			

*Based on the clinical experience of Susan Swedo, M.D., Miroslav Kovacevic, M.D., Beth Latimer, M.D., and James Leckman, M.D., with the help of Diana Pohlman, Keith Moore and many other parents. **Six Obsessive-compulsive symptoms domains are presented. Rate all of them. However, on the above table only enter the score of the most severe domain (times 5; 0-25).***Seven Associated symptom domains are presented. Rate all of them. However, for each domain one or more symptom sets are listed. On the above table, only enter the score of the most severe symptom set for each domain (0-5).

Date:

Name:

SYMPTOM SEVERITY RATING SCALE (use these anchor points for each of the symptoms)

**Severity** ***(rate each of the symptoms listed on the following pages for the past week)***	
**NONE** No evidence of specific symptoms and behaviors	**0**
**MINIMAL** Specific symptoms and/or behaviors are present but are only evident occasionally and not a major source of difficulty.	**1**
**MILD** Specific symptoms and/or behaviors are present during the past week, and are episodically a source of some distress and difficulty.	**2**
**MODERATE** Specific symptoms and/or behaviors are present every day and are a source of distress and difficulty.	**3**
**SEVERE** Specific symptoms and/or behaviors are present every day and are severe resulting in a great deal of distress and difficulty.	**4**
**EXTREME** Specific symptoms and/or behaviors are always present and are extremely severe resulting in an extreme degree of distress and difficulty.	**5**

If multiple time points will be rated on this form, please use the following indicators:

"**B**" = Symptom severity one week ***Before*** the onset of the first episode of illness

"**O**" = Symptom severity during the week following the initial ***Onset*** of symptoms

"**C**" = ***Current*** symptom severity during the past week

Date:

Name:

Informant:

I. CORE Obsessive-compulsive Symptoms (circle and rate ALL symptoms that have been present in the past week). Use the "BOC" indicators if multiple time points are being scored (see p. 2).

Obsessive-compulsive symptoms	0 = Absent	1 = Minimal	2 = Mild	3 = Moderate	4 = Severe	5 = Extreme
Intrusive and persistent obsessional worries (anxieties) about dirt and germs and related washing compulsions (circle obsessions and/or compulsions)						
Intrusive and persistent obsessional worries (anxieties) about harm to self or others and related compulsions; a need to tell or confess (this symptom domain may be closely related to separation worries, but rate both if both are present						
Intrusive and persistent obsessional worries (anxieties) about sexual or religious thoughts or behaviors and related rituals and compulsions						
Intrusive obsessional worries about symmetry and related compulsions: ordering, counting, *or* arranging; a need to touch, tap or rub, or a need for things to feel, look, *or* sound 'just right'						

**Obsessive-compulsive symptoms**	**0 = Absent**	**1 = Minimal**	**2 = Mild**	**3 = Moderate**	**4 = Severe**	**5 = Extreme**
Intrusive and persistent obsessional worries (anxieties) about collecting and hoarding						
Restrictive and/or avoidant food intake symptoms; Eating or feeding disturbance (including but not limited to apparent lack of interest in eating or food; avoidance based on the sensory characteristics of food; or concern about aversive consequences of eating) resulting in a refusal to eat (atypical anorexia) or a marked decrease in food intake						
Miscellaneous. The need to know or remember; Fear of saying certain things; Fear of not saying just the right thing; Intrusive (non-violent) images; Intrusive sounds, words, music or numbers; Need to repeat activities (e.g. in/out of a doorway, up/down from chair); The need to involve another person (usually a parent) in ritual (e.g. asking a parent to repeatedly answer the same question; Mental rituals other than checking / counting; Excessive list making; Other (describe) ______________________ ______________________						
**Severity of all the above Obsessive-compulsive symptoms (**over the past week) Five times this rating [0-25] should be entered on p. 1						

II. ASSOCIATED SYMPTOMS (circle and rate ALL symptoms that have been present in the past week). Use the "BOC" indicators if multiple time points are being scored (see p. 2).

**1. Anxiety symptoms**	**0 = Absent**	**1 = Minimal**	**2 = Mild**	**3 = Moderate**	**4 = Severe**	**5 = Extreme**
Separation anxiety – need to maintain proximity to person, a familiar location, or a thing						
General anxiety						
Unfounded irrational fears and/or phobias						
Panic episodes						

**2. Emotional lability, depression,**	**0 = Absent**	**1 = Minimal**	**2 = Mild**	**3 = Moderate**	**4 = Severe**	**5 = Extreme**
Emotional lability – mood swings - moodiness						
Depression with or without suicidal or self-injurious thoughts						

**3. Increased irritability or aggressive behavior**	**0 = Absent**	**1 = Minimal**	**2 = Mild**	**3 = Moderate**	**4 = Severe**	**5 = Extreme**
Increased irritability; defiant/ irrational demands; reactive aggressive behavior, temper tantrums; rage attacks						

**4. Behavioral regression**	**0 = Absent**	**1 = Minimal**	**2 = Mild**	**3 = Moderate**	**4 = Severe**	**5 = Extreme**
Behavioral regression ("baby talk," behavior atypical for actual chronological age)						
Change in personality						

**5. School performance Concentration/ Learning**	**0 = Absent**	**1 = Minimal**	**2 = Mild**	**3 = Moderate**	**4 = Severe**	**5 = Extreme**
Difficulties in attention, concentration or learning – unable to concentrate or a clear problem with immediate or short term memory						
Loss of academic skills – especially math or in reading or writing						
Confusion						

**6.A. Sensory symptoms**	**0 = Absent**	**1 = Minimal**	**2 = Mild**	**3 = Moderate**	**4 = Severe**	**5 = Extreme**
Heightened sensitivity to light, the way things “feel” (tags or labels) or “sound” or other sensory stimuli – such as smell or taste; a need to touch things in a specific way; how things “look” including spatial distortion (eg, objects appear closer than they actually are)						

**6.B. Hallucinations.**	**0 = Absent**	**1 = Minimal**	**2 = Mild**	**3 = Moderate**	**4 = Severe**	**5 = Extreme**
Visual or auditory hallucinations						

6.C. Motor symptoms	0 = Absent	1 = Minimal	2 = Mild	3 = Moderate	4 = Severe	5 = Extreme
Dysgraphia (loss of ability to draw, copy figures and/or write letters)						
Motoric hyperactivity and/or adventitious movements - kicking, spitting, flailing, rolling, or stomping (do not rate tics here); unable to stay still						
Piano playing finger movements						
Simple motor tics or vocal tics (grunting, squeaking, etc)						
Complex motor or vocal tics including; spitting, obscene words or actions, repeating words or actions changes in rate or pitch of speech						

7.A. Urinary symptoms	0 = Absent	1 = Minimal	2 = Mild	3 = Moderate	4 = Severe	5 = Extreme
Urinary frequency or increased urge to urinate; daytime or night; inability to urinate						

**7.B. Sleep disturbance - Fatigue**	**0 = Absent**	**1 = Minimal**	**2 = Mild**	**3 = Moderate**	**4 = Severe**	**5 = Extreme**
Sleep problems (lengthy bedtime rituals, insomnia, inability to sleep; hypersomnia, nightmares)						
Extreme tiredness or fatigue						

**7.C. Dilated pupils**	**0 = Absent**	**1 = Minimal**	**2 = Mild**	**3 = Moderate**	**4 = Severe**	**5 = Extreme**
Dilated pupils –“terror stricken look”						

Name:

Informant:

III. Impairment Rating

Use the "BOC" indicators if multiple time points are being scored (see p. 2).

**IMPAIRMENT *(past week)***		
**NONE**	**0**	
**MINIMAL** Symptoms associated with subtle difficulties in self-esteem, family life, social acceptance, or school or job functioning (infrequent upset or concern about tics *vis a vis* the future, periodic, slight increase in family tensions because of symptoms; friends or acquaintances may occasionally notice or comment about symptoms in an upsetting way).	**10**	
**MILD** Symptoms associated with minor difficulties in self-esteem, family life, social acceptance, or school functioning.	**20**	
**MODERATE** Symptoms associated with some clear problems in self-esteem family life, social acceptance, or school or job functioning (episodes of dysphoria, periodic distress and upheaval in the family, frequent teasing by peers or episodic social avoidance, periodic interference in school performance because of PANS symptoms.	**30**	
**SEVERE** Symptoms associated with major difficulties in self-esteem, family life, social acceptance, or school functioning.	**40**	
**EXTREME** Symptoms associated with extreme difficulties in self-esteem, family life, social acceptance, or school functioning (severe depression with suicidal ideation, disruption of the family (separation/divorce, residential placement), disruption of social ties - severely restricted life because of social stigma and social avoidance, removal from school).	**50**	
