# Supplementary material for: Evaluation of Intravenous Immunoglobulin in Pediatric Acute-Onset Neuropsychiatric Syndrome
Source: J Child Adolesc Psychopharmacol. 2021 Mar 15;31(2):118–28. doi: 10.1089/cap.2020.0100 (PMC7984935; doi:10.1089/cap.2020.0100)
Supplement: Supplemental data [file Supp_App2.pdf]

PANS Questionnaire

Parent-Rated Symptom Severity

Subject Identifier: ____________________ Date:____________________

INSTRUCTIONS:
This form is for you to rate your child's symptoms related to PANS at each study visit as part of the clinical trial. You will be able to review the previous visit to note if there have been any changes in any of the behaviors. The ratings are to show any worsening of the condition or improvement. The column for previous behavior would be checked if the child had this symptom prior to PANS. For example, if your child had attention issues before the diagnosis of PANS, please check the box for previous behavior and then rate the severity.

As well, we would like to capture your ratings of the initial on-set of the PANS symptoms. At the screening visit, please fill out one for the initial onset and one for how they are at the time of the visit. Please check the box if you feel that the symptoms at each visit are 'spiking' (exacerbation) or if the symptoms have reduced to be in more of a 'remission' phase (remission). Feel free to write any added comments or symptoms that are not in the form that you feel are important.

☐Screening ☐Infusion # _____ ☐ End of study

Parent-Rated Syndrome Status:

☐ Initial – historical ☐ Exacerbation ☐ Remission

Does your child experience:	None	Mild	Moderate	Severe	Extreme	Previous behavior
1. Separation anxiety	0	1	2	3	4	
2. Irrational fears or worries	0	1	2	3	4	
3. Specific phobias - ____________	0	1	2	3	4	
4. Sleep disturbances	0	1	2	3	4	
5. Difficulty falling asleep	0	1	2	3	4	
6. Difficulty staying asleep	0	1	2	3	4	
7. Waking too early	0	1	2	3	4	
8. Bedtime fears	0	1	2	3	4	
9. Nightmares	0	1	2	3	4	

PANS Questionnaire

Parent-Rated Symptom Severity

Subject Identifier: ____________________ Date:_____________________

Does your child experience:	None	Mild	Moderate	Severe	Extreme	Previous behavior
10. Increase in frequency of urination	0	1	2	3	4	
11. Urinary urgency	0	1	2	3	4	
12. Enuresis - bed wetting	0	1	2	3	4	
13. Sensory defensiveness	0	1	2	3	4	
14. Sensitive to light	0	1	2	3	4	
15. Sensitive to noises	0	1	2	3	4	
16. Sensitive to smells	0	1	2	3	4	
17. Sensitive to textures - touch	0	1	2	3	4	
18. Sensitive to clothing	0	1	2	3	4	
19. Need to touch (feel) specific items or textures	0	1	2	3	4	
20. Change in food intake or eating behaviors	0	1	2	3	4	
21.Anorexic behavior	0	1	2	3	4	
22.Body-image distortion	0	1	2	3	4	
23. Sensitive to food texture	0	1	2	3	4	
24. Fear of choking	0	1	2	3	4	
25. Fear of contamination	0	1	2	3	4	
26. Irritability	0	1	2	3	4	
27. Agitation	0	1	2	3	4	
28. Depressive state	0	1	2	3	4	
29. Oppositional behaviors	0	1	2	3	4	
30. Defiant behavior	0	1	2	3	4	
31.Aggressive behaviors	0	1	2	3	4	
32.Fear of harming others	0	1	2	3	4	
33. Fear of harm to self	0	1	2	3	4	
34. Self-injurious behaviors	0	1	2	3	4	
35. Mood swings - emotional lability	0	1	2	3	4	
36. Obsessive compulsive behaviors (OCD)	0	1	2	3	4	
37. OCD behaviors at home	0	1	2	3	4	
38. OCD behaviors in school	0	1	2	3	4	
39. OCD behaviors with peers	0	1	2	3	4	
40. Excessive ritualized hand-washing	0	1	2	3	4	

PANS Questionnaire

Parent-Rated Symptom Severity

Subject Identifier: ____________________ Date: ____________________

Does your child experience:	None	Mild	Moderate	Severe	Extreme	Previous behavior
41. Excessive cleaning	0	1	2	3	4	
42. Excessive concern with illness or disease	0	1	2	3	4	
43. Repeated rituals	0	1	2	3	4	
44. Checking compulsion	0	1	2	3	4	
45. Inattention	0	1	2	3	4	
46. Hyperactivity	0	1	2	3	4	
47. Impulsivity	0	1	2	3	4	
48. Motor tics	0	1	2	3	4	
49. Abnormal hand or finger movements	0	1	2	3	4	
50. Increase in clumsiness	0	1	2	3	4	
51. Change in gait	0	1	2	3	4	
52. Behavioral regression	0	1	2	3	4	
53. Language regression	0	1	2	3	4	
54. Decline in handwriting	0	1	2	3	4	
55. Decline in school performance	0	1	2	3	4	
56. Loss of math skills	0	1	2	3	4	
57. Decline in artistic skills	0	1	2	3	4	
58. Decline in school attendance	0	1	2	3	4	

COMMENTS:

____

____

____

____

____

____

____

____
